# Barriers and delays in access to abortion care: a cross-sectional study of people traveling to obtain care in England and the Netherlands from European countries where abortion is legal on broad grounds

**DOI:** 10.1186/s12978-023-01729-2

**Published:** 2024-01-15

**Authors:** Alexandra Wollum, Silvia De Zordo, Giulia Zanini, Joanna Mishtal, Camille Garnsey, Caitlin Gerdts

**Affiliations:** 1 Ibis Reproductive Health, 1111 Broadway, Oakland, CA 94607 USA; 2 Department of Anthropology, University of Barcelona, Montalegre 6-8, 08001 Barcelona, Spain; 3 Department of Philosophy and Cultural Heritage, Ca’ Foscari University of Venice, Venice, Italy; 4 Department of Anthropology, University of Central Florida, 4297 Andromeda Loop, Orlando, FL 32816 USA

**Keywords:** Abortion, Europe, Travel, Delays, Financial barriers, Health system barriers

## Abstract

**Introduction:**

This study characterized the extent to which (1) financial barriers and (2) abortion care-seeking within a person’s country of residence were associated with delays in abortion access among those travelling to England and the Netherlands for abortion care from European countries where abortion is legal on broad grounds in the first trimester but where access past the first trimester is limited to specific circumstances.

**Methodology:**

We drew on cross-sectional survey data collected at five abortion clinics in England and the Netherlands from 2017 to 2019 (n = 164). We assessed the relationship between difficulty paying for the abortion/travel, acute financial insecurity, and in-country care seeking on delays to abortion using multivariable discrete-time hazards models.

**Results:**

Participants who reported facing both difficulty paying for the abortion procedure and/or travel and difficulty covering basic living costs in the last month reported longer delays in accessing care than those who had no financial difficulty (adjusted hazard odds ratio: 0.39 95% CI 0.21–0.74). This group delayed paying other expenses (39%) or sold something of value (13%) to fund their abortion, resulting in ~ 60% of those with financial difficulty reporting it took them over a week to raise the funds needed for their abortion. Having contacted or visited an abortion provider in the country of residence was associated with delays in presenting abroad for an abortion.

**Discussion:**

These findings point to inequities in access to timely abortion care based on socioeconomic status. Legal time limits on abortion may intersect with individuals’ interactions with the health care system to delay care.

## Plain Language Summary

This paper explores delays in accessing abortion care associated with financial and medical system barriers. We focus on residents of countries in Europe where abortion is available on broad grounds in the first trimester seeking abortion care outside of their country of residence. This study demonstrates an association between difficulty covering abortion costs for people facing financial insecurity and in-country care seeking and delays in accessing abortion abroad. Policy barriers, medical system barriers, as well as financial barriers may interact to delay access to care for people in European countries with broad grounds for abortion access in the first trimester but restrictions thereafter, especially for people later in pregnancy.

## Background

Even in contexts where abortion is available on broad legal grounds (i.e., available on request or on broad social and economic grounds), barriers exist to seeking wanted abortion care. Financial, procedural, informational, and social barriers limit access to abortion services and compel people to travel for services, causing unique burdens such as lost wages, increased costs related to childcare and transportation, increased need to disclose seeking services, and delayed care [1–7]. Delays in accessing abortion care cause procedures to take place later in pregnancy—increasing both the cost of care and the risk of complications [8].

The literature exploring financial and logistical barriers to abortion care in Europe, especially in countries where abortion is broadly legal in the first trimester but restricted to specific circumstances thereafter, is limited [6, 9, 10]. Although abortion is legal in nearly all European countries (with the exception of Poland, Malta, and until 2018 and 2019 the Republic of Ireland and Northern Ireland respectively), restrictions on timing, permitted reasons, and waiting periods vary across countries [11, 12]. In most countries in Europe including those included in the present analysis (Italy, France,^1^ Germany, Belgium, Austria, and Denmark) abortion is permitted on broad grounds in the first trimester, but highly restricted thereafter. After the first trimester, abortion is only permitted on certain grounds, most commonly in cases that a pregnant person’s life or health is endangered, in cases of fetal anomalies, or rape and incest. Other laws include mandatory waiting periods and mandatory counseling [14, 15]. Provider shortages (due to belief-based denial or lack of second trimester training) and limited service provision outside of urban areas also create barriers to care [11, 16–25]. Because of such barriers, pregnant people may be forced to travel to other regions of their country or to another country to seek abortion services, especially if they are seeking services later in pregnancy [26]. However, the evidence surrounding travel for abortion in Europe has primarily focused on contexts in which abortion is highly restricted throughout pregnancy and little is known about the experiences of those travelling from countries where abortion is available on broad grounds in the first trimester [6].

Because England, the Netherlands, and Spain are among the only European countries with simplified legal access to care after the first trimester and because of their proximity to many European countries, many people needing abortion services past the first trimester travel to these countries to seek later abortion care [2, 10, 27, 28]. Cross-country travel, however, has been associated with delays in access to abortion care [29]. Travelling for abortion care incurs costs which literature has documented to be burdensome [2, 27]. Additionally, because abortion is broadly legal in the first trimester in many European countries, people seeking abortion may look to local resources prior to seeking care abroad. While a growing mixed-methods literature has explored people’s experiences with travel for abortion in Europe [10, 26, 30–32], little is known about how cost and in-country care seeking are associated with delays in care. For these reasons, we investigate two potential factors that may delay care seeking including (1) care-seeking within the country of residence and (2) financial barriers to care among those travelling to England and the Netherlands for abortion care. Understanding whether these factors delay access to abortion care for this understudied population is important to inform policies and interventions to increase access to timely abortion care.

## Materials and methods

For this analysis, we draw on data from a multi-country, 6-year, mixed-methods study on barriers to legal abortion and travel for abortion in Europe, funded by the European Research Council. The study aimed to assess the impact of legal, procedural, and social barriers to abortion care, and to document and explore the experiences of women and pregnant people^2^ who travel abroad to seek abortion services in England, the Netherlands, and Spain as well as those who travel domestically within their country of residence in France, Italy and Spain. This analysis focuses on the quantitative survey data collected among those travelling from countries where abortion is legal on broad grounds to England and the Netherlands (n = 204). We exclude data from Spain because we recruited few participants that travelled from abroad. See Table 1 for a list of abortion laws in countries in which participants resided. We excluded those traveling from restrictive contexts (Poland, Malta, and at the time of our data collection the Republic of Ireland) from the analysis for two reasons. First, in-country care seeking in restrictive contexts may be less relevant and second, the population of travelers from restrictive contexts is distinct. Those traveling from restrictive contexts primarily sought care during their first trimester while those recruited participants from settings where abortion is legal on broad grounds were largely in the second trimester of their pregnancy.

**Table 1:** Selected Abortion laws in countries from which participants travelled at time of study (2017–2019)*

**Country**	Description of law
Germany	Abortion legal up to 12 weeks after conception on request or in the case of medical indications (serious physical or mental health problems of the pregnant person) or criminal indications (rape). Abortion after 12 weeks allowed on medical or criminal grounds only [54] Mandatory counseling [14, 55] Mandatory 3 day waiting period [54] Abortion providers banned from providing information on abortion or advertising abortion services [45]
France	Abortion legal up to 12 weeks upon request.^^^ Abortion after 12 weeks allowed for medical reasons including danger to the pregnant person’s health or serious and incurable fetal conditions [56] Mandatory counseling for those under 18 years old [57]
Italy	Abortion legal for first 90 days of pregnancy^#^ on broad grounds (a serious threat to the mother’s physical or psychological health, for her health situation, her economic, social or familial conditions, the circumstances of conception, or anomalies or malformations of the foetus). After 90 days only medical exceptions for access to abortion including danger to pregnant person’s life and pathologies affecting the woman or foetal malformation that determine a serious danger for her health (physical or mental) [58] Minors require parental consent or judicial bypass Mandatory 7 day waiting period [15]
Belgium	Abortion legal up to 12 weeks on the grounds of “distress and emergency” after which abortion legal only if serious risk to pregnant person’s life or the fetus has severe malformations [59] Mandatory 6 day waiting period [15] Mandatory counseling on alternatives to abortion [59]
Austria	Legal in the first three months of pregnancy on demand. After 16 weeks legal in case of physical or mental threat to pregnant person, severe fetal malformations, or if pregnant person is under 14 [60, 61]
Denmark	Legal up to 12 weeks of pregnancy on demand. After 12 weeks abortion must be approved by a council. Can be considered in cases of pregnant person’s life or health in danger or due to existing physical or mental illness, rape or incest, severe fetal malformation, demonstrated “inability to care” for child (61, 62) -Parental consent required for minors[64]

*Gestational age limits as reported by each country’s law

^^^France provider and medical authorities interpreted the 12-week limit as equivalent of 14 weeks after last menstrual period

^#^Italian medical and legal authorities interpret this to mean from last menstrual period

We selected three clinics run by the British Pregnancy Advisory Service (BPAS) in England and two abortion clinics in the Netherlands for recruitment that had the largest number of non-residents who obtained abortion care at the respective clinics in the years preceding the launch of the study. Abortion patients were eligible to participate if they were 18 years of age or older, had travelled from another European Union country to seek abortion care, and were proficient in French, Italian, English, Dutch, German or Spanish. Eligible individuals were identified by an on-site researcher and/or clinic staff and provided with a study information sheet upon their arrival to the clinic. Those interested could complete an anonymous, self-administered, tablet-based questionnaire at the clinic at any time before starting the abortion procedure, or remotely, via phone or internet, after going back to their countries of residence. Only two participants participated remotely after their procedures. These participants were excluded from this analysis. The survey covered topics such as sociodemographic information, reproductive histories, care-seeking trajectory, barriers faced in accessing abortion services in the country of residence, travel and cost in care seeking, and reasons for and experiences in seeking abortion care out of the country of residence. Recruitment spanned July 2017 to March 2019. We completed data collection prior to the withdrawal of the United Kingdom from the European Union; however, data collection started after the vote on the referendum to approve the withdrawal in 2016. We aimed to recruit 200 respondents across the full study in England and 200 respondents in the Netherlands to have sufficient power for the study’s main proposed analyses which involved comparing respondents by country of residence legal context. We aimed to recruit as many respondents as possible within each participating clinic until our country-specific sample size was reached; however, we recruited fewer respondents than anticipated.

### Measures

Our analysis aims to characterize the extent to which (1) financial barriers and (2) abortion care seeking within a person’s country of residence are associated with delays in abortion access.

#### Outcome

The main outcome of the analysis was delays in accessing abortion care. We defined delays as the number of weeks between when the respondent considered abortion and the day they completed the survey. We excluded respondents missing this value from the analysis (n = 24).

#### Predictors of interest

We evaluate two specific predictors of interest: (1) the difficulty of covering the costs of travel and the abortion procedure and (2) whether the respondent had contacted or visited any abortion providers in their country of residence before coming to the clinic where they were surveyed. For the first predictor, we created a composite, binary variable to summarize the responses from the two questions “How easy or difficult would you say it was for you to cover the cost of travel, not including the abortion itself?” and “How easy or difficult would you say it was for you to cover the cost of the abortion procedure?” We combined those who responded that it was “very easy” or “somewhat easy” to cover both the cost of the abortion and travel in one group representing no difficulty in covering costs. We considered those who said the cost of travel, the cost of the abortion, or both costs were “somewhat difficult” or “very difficult” to cover to have some difficulty in covering costs. This measure relies on the participant’s assessment of the difficulty or ease with which they were able to cover the cost of the abortion and travel.

We created a second categorical predictor to test the intersection of difficulty paying for the abortion and travel and other basic living costs. To assess whether respondents had sufficient funds to cover basic living costs in the past month, we assessed responses to the question: “During the past month, would you say you had enough money to meet your basic living needs such as food, housing and transportation?” An answer of “all the time” or “most of the time” was categorized as having sufficient funds to cover basic living costs, an answer of “some of the time”, “rarely”, or “never” was categorized as having insufficient funds to cover basic living costs. Using this question we created a predictor with the following categories: (1) no difficulty paying for the abortion or travel **AND** sufficient funds to cover basic living costs (2) some difficulty paying for the abortion or travel **AND** sufficient funds to cover basic living costs (3) no difficulty paying for the abortion or travel **AND** not having sufficient funds to cover basic living costs and (4) some difficulty paying for the abortion or travel **AND** not having sufficient funds to cover basic living costs. The one participant who fell into the third category was excluded from analysis. These groups are referred to as “highest means,” “mixed means,” and “lowest means,” respectively. Responses were missing for n = 31 participants.

To assess whether there were any differences in delays based on in-country care seeking, we used a binary measure of whether a respondent reported contacting and/or visiting a provider in their country of residence. We ran a sensitivity analysis using a secondary measure of in-country care seeking. This measure categorized respondents in three categories: no contact and no visit to providers, contacted providers only, and visited providers. Responses were missing for three participants.

We excluded observations that were missing any of the main predictors from our analyses.

#### Covariates

We considered possible confounders of the relationship between the two predictors of interest and the outcome based on their theorized associations between financial difficulty, in-country care seeking, and delays in care. We controlled for age of the respondent (categorical variable 18–24, 25–34, 35+); a categorical measure of parity (no children, one-two children, and 3+ children); country of residence; gestational age of the pregnancy at the time of the survey; reported difficulty of the abortion decision; whether the participant had tried anything on their own to end the pregnancy; history of prior abortion; social support in their abortion decision-making process; employment status (full time employment (> 32 h/week), part time employment, student, or other (unemployed, unable to work, homemaker)); and the time it took to travel to the abortion clinic (≤ 2 h, > 2–4 h, > 4–6 h, > 6–8 h, 8+ h). We also controlled for the difficulty covering the cost of the abortion in the analysis of in-country care-seeking.

We also examined secondary measures of cost and financial experiences of abortion and travel asked in our survey including logistics organized for the abortion appointment and travel, actions taken to cover the cost of the abortion, length of time needed to raise money for the cost of travel and the abortion, and cost-related reasons for delays in abortion.

### Analysis

We used Stata v15 SE to conduct quantitative analyses. We calculated descriptive frequencies and bivariate associations for the outcome and key predictors. We stratified descriptive results about cost and financial experiences by the difficulty of paying for the procedure and ability to cover basic living expenses. In order to test the associations between the predictors of interest and delays in access to care controlling for potential confounders, we constructed multivariate discrete-time hazards models using the weeks of delay as the unit of time and logit link. In this analysis, shorter “survival” or having received an abortion earlier after considering abortion is the preferred outcome. Standard errors were clustered by respondent. The clinic at which the patient received services is controlled for as a fixed effect in the model. We tested the sensitivity of the results to the handling of missing data by running a model using pairwise deletion for each model instead of using casewise deletion with any missing main predictor. We also coded a “missing” category for the main predictors and re-ran the model with the re-coded predictors.

### Ethical approval

This phase of the study received ethical approval from the European Research Council Ethics Committee, the BPAS Research & Ethics Committee, the Tilburg University Ethics Committee, and the University of Barcelona Bioethics Committee.

## Results

The main analysis included a total of 164 participants. The majority of respondents (86%) sought abortion services in the Netherlands while 14% were recruited in England (Table 2). Over half of the sample resided in Germany, and a quarter lived in France. The rest of the sample lived in Italy (8.5%), Belgium (6.7%), or another country (7.3%) within Europe where abortion is legal on broad grounds. Similar proportions of participants were between 18 and 24 years old and 25 and 34 at the time of the survey (42.1% and 44.5% respectively). The majority of participants did not have children (62.8%), had completed some university or more (61.6%), and had enough money all the time or most of the time to meet their basic living needs in the past month (76.8%). At the time of the survey, the majority of respondents reported gestational ages between 13 and 20 weeks with a mean gestational age of 17.8. The majority of respondents had to travel over 2 h with approximately 30% reporting they traveled for over 6 h. The main reasons for travel included that it was too late to have an abortion in their country of residence (81%) and that abortion was not legal in my their country of residence in their situation (7%) (data not shown).

**Table 2:** Sample characteristics, difficulty paying for the abortion/travel, in-country care seeking, and delays in accessing care (n = 164)

	Full sample n (%)	% any difficulty covering payment for abortion or travel	% contacted or visited abortion provider in country of residence	Weeks between considering abortion and survey
Full sample	164 (100%)	67.7	47.6	4.2
Country of abortion services				
The Netherlands	141 (86%)	66.7	44.0	4.2
England	23 (14%)	73.9	69.6	4.3
Country of residence				
France	40 (24.4%)	72.5	40.0	4.6
Italy	14 (8.5%)	57.1	78.6	4.4
Germany	87 (53.0%)	65.5	39.1	4.1
Belgium	11 (6.7%)	72.7	63.6	2.7
Other	12 (7.3%)	75.0	83.3	4.7
Age				
18–24	69 (42.1%)	71.0	42.0	5.1
25–34	73 (44.5%)	65.8	54.8	3.7
35 or above	22 (13.4%)	63.6	40.9	3.2
Children				
0	103 (62.8%)	68.0	44.7	4.3
1–2	49 (29.9%)	67.3	49.0	4.1
3+	12 (7.3%)	66.7	66.7	3.8
Educational attainment				
Primary School	13 (7.9%)	76.9	69.2	7.5
Secondary School	47 (28.7%)	74.5	44.7	4.3
Some university	39 (23.8%)	66.7	41.0	4.1
University	44 (26.8%)	61.4	54.5	3.6
Post graduate	18 (11.0%)	61.1	33.3	3.3
Prefer not to answer	3 (1.8%)	66.7	66.7	2.3
Marital status				
Married or in a civil partnership/cohabitating	83 (50.6%)	61.4	43.4	4.2
Single, separated, or divorced	72 (43.9%)	75.0	52.8	4.3
Other	5 (3.1%)	60.0	40.0	3.8
Prefer not to answer	4 (2.4%)	75.0	50.0	2.3
Employment status				
Employed full time	66 (40.2%)	65.2	47.0	3.6
Employed part time	16 (9.8%)	75.0	37.5	5.6
Freelancer	8 (4.9%)	50.0	37.5	2.9
Student	39 (23.8%)	71.8	46.2	2.9
Other/unemployed	31 (18.9%)	71.0	58.1	6.9
Missing	4 (2.4%)	50.0	50.0	2.3
Prior abortion				
No	126 (76.8%)	65.1	46.0	4.2
Yes	37 (22.6%)	78.4	51.4	4.3
Prefer not to answer	1 (0.6%)	0.0	100.0	2.0
Did something on own to try to end the pregnancy				
No	152 (92.7%)	67.1	46.7	4.2
Yes	9 (5.5%)	66.7	55.6	4.7
Prefer not to answer	3 (1.8%)	100.0	66.7	3.7
Gestational age at time of seeking services				
1–12 weeks	7 (4.3%)	14.3	42.9	2.3
13–20 weeks	127 (77.4%)	71.7	47.2	4.1
More than 20 weeks	29 (17.7%)	62.1	48.3	5.1
Prefer not to answer	1 (0.6%)	100.0	100.0	3.0
During the past month would you say you had enough money to meet your basic living needs?				
All the time	72 (43.9%)	43.1	43.1	3.6
Most of the time	54 (32.9%)	81.5	42.6	3.7
Sometimes/rarely/never	32 (19.5%)	97.1	61.8	6.5
Missing	6 (3.7%)	75.0	100.0	2.5
How would you describe how you reached your decision to have an abortion?				
Very or somewhat easy	67 (40.9%)	61.2	43.3	3.9
Neither easy nor difficult	27 (16.5%)	66.7	48.1	4.0
Very or somewhat difficult	68 (41.5%)	73.5	51.5	4.6
Prefer not to answer	2 (1.2%)	100.0	50.0	2.5
Felt supported by family, friends, and/or partner				
No	22 (13.4%)	77.3	54.5	4.9
Yes	141 (86.0%)	66.0	46.8	4.1
Missing	1 (0.6%)	100.0	0.0	2.0
Difficulty of travel abroad				
Very easy	20 (12.2%)	45.0	60.0	3.4
Somewhat easy	89 (54.3%)	62.9	40.4	4.4
Somewhat difficult	42 (25.6%)	83.3	54.8	3.1
Very difficult	12 (7.3%)	83.3	58.3	7.2
Missing	1 (0.6%)	100.0	0.0	12.0
Travel time to clinic				
Less than or equal to 2 h	22 (13.4%)	68.2	50.0	3.8
> 2 to 4 h	48 (29.3%)	64.6	47.9	3.8
> 4 to 6 h	40 (24.4%)	72.5	37.5	4.5
> 6 to 8 h	25 (15.2%)	64.0	52.0	3.7
More than 8 h	25 (15.2%)	68.0	52.0	5.6
Missing	4 (2.4%)	75.0	75.0	2.8

About two-thirds of the sample had some difficulty covering the cost of the abortion procedure and/or the travel (Table 2). Forty-eight percent of the sample contacted or visited an abortion provider in their country of residence before seeking services abroad (Table 2, column 3). Of these respondents, 21% contacted a provider but did not visit any provider in person while 79% visited a provider in person in their country of residence.

On average, approximately 4.2 weeks elapsed between when participants considered abortion and when they were surveyed at the clinic when presenting for abortion care (Table 2, column 4). In the sample, the weeks elapsed ranged from a minimum of zero weeks to a maximum of 21 weeks.

### Financial barriers

**Fig. 1:**
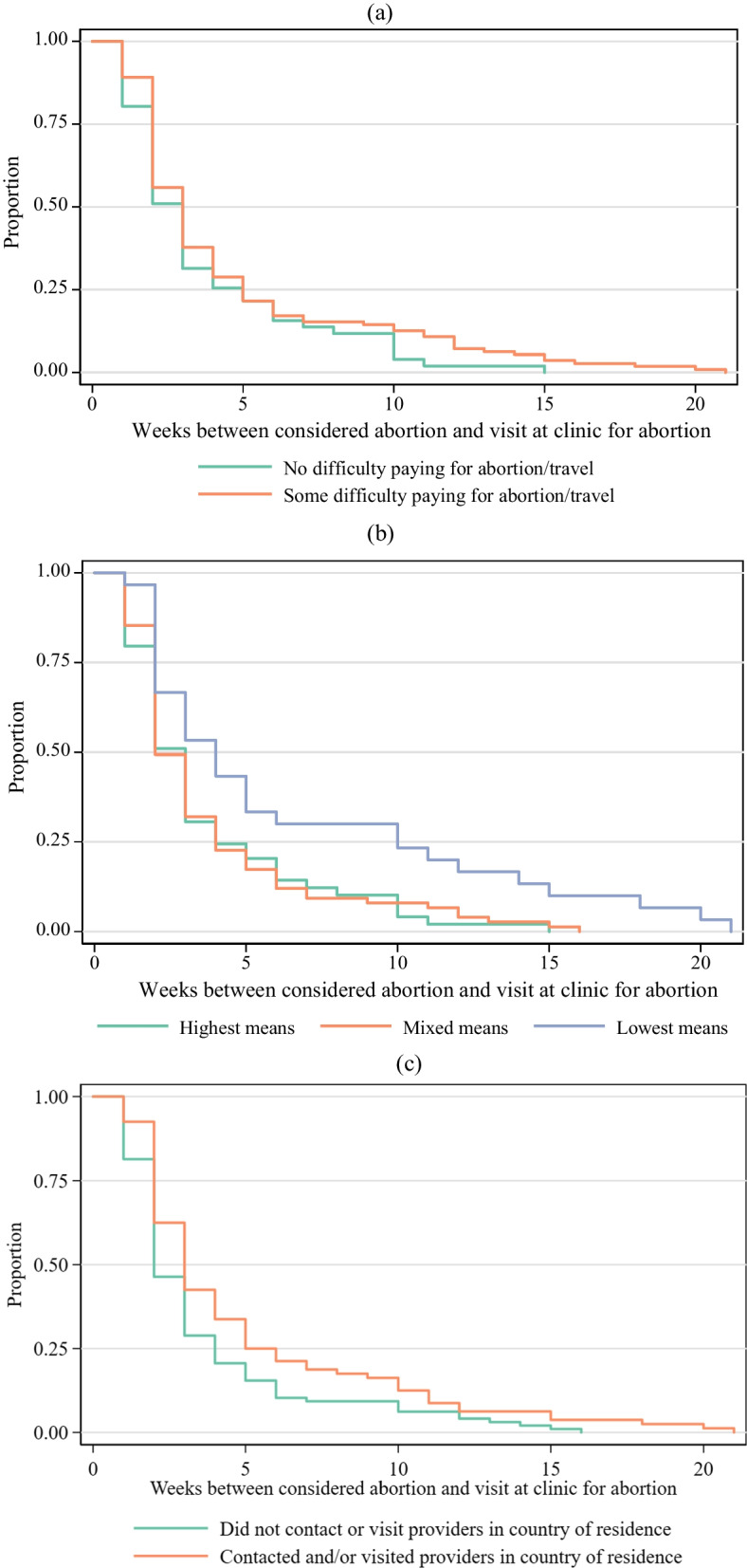

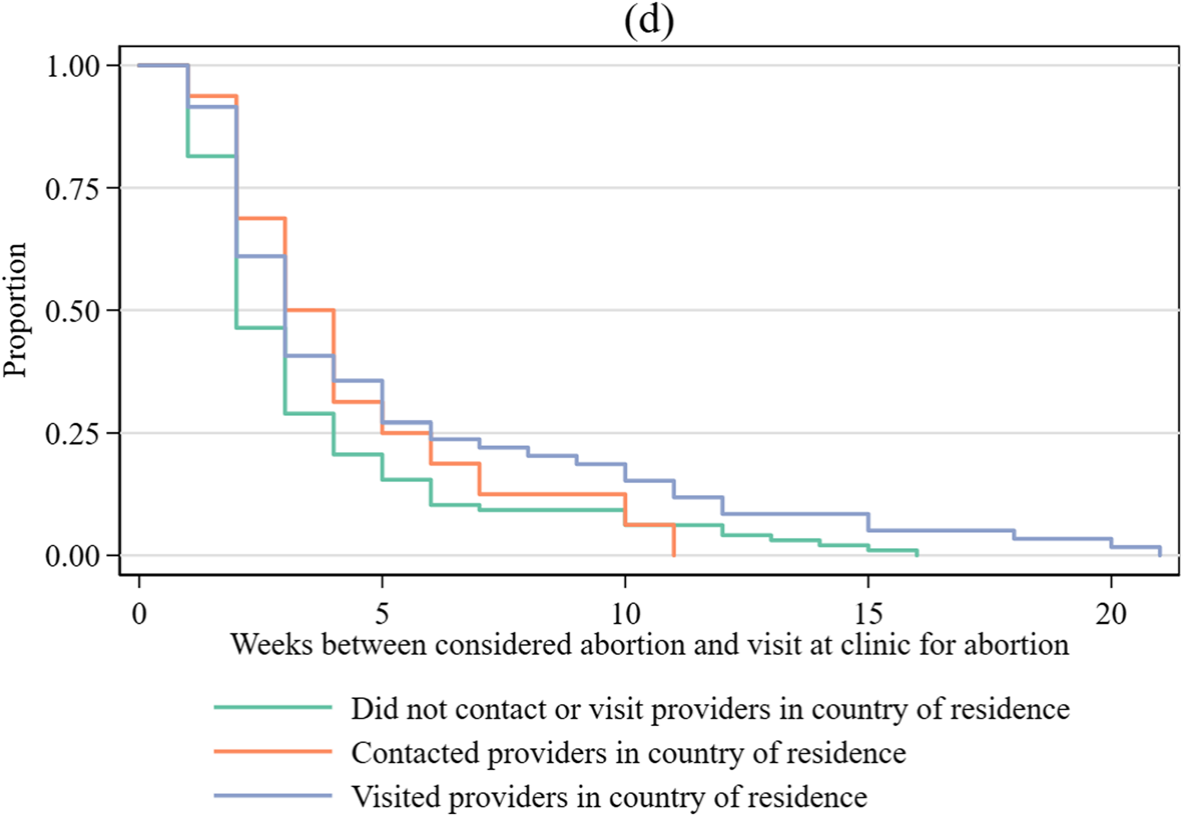
Cumulative proportion of participants that had presented at abortion clinic abroad from time since first considered abortion among those from contexts where abortion is legal on broad grounds (n = 164). **a** Difficulty paying for abortion and/or travel. **b** Difficulty paying for abortion and/or travel and availability of funds to cover basic living costs in past 30 days. **c** Contacted or visited abortion providers in country of residence. **d** No in-country care seeking, contact only, and visited abortion provider in country of residence. Highest means represent those who had no difficulty paying for abortion procedure and travel and had funds to cover basic living expenses in the past month “all the time” or “most of the time.” Mixed means represent those who had some difficulty paying for abortion procedure and travel and had funds to cover basic living expenses in the past month “all the time” or “most of the time.” Those with lowest means had some difficulty paying for abortion procedure and travel and had funds to cover their basic living expenses in the past month “sometimes”,  “rarely,” or “never"

Comparing time to presentation at the clinic by difficulty paying for the abortion or travel alone, approximately 50% of both those who reported no difficulty covering costs and those who reported some difficulty covering costs had presented at the clinic where they completed the survey by at least 3 weeks after considering the abortion (Fig. 1a).

Considering the extent to which someone is able to cover their basic living expenses *and* had difficulty covering the cost of the abortion and/or travel (Fig. 1b), those with the lowest means reported longer delays between when they considered abortion and when they presented at the clinic. Among those with the highest means and those with mixed means, 50% of participants had presented at the clinic abroad by two to three weeks after considering abortion and 75% had presented by 4 weeks. Among those with the lowest means, 50% of participants had presented at 4 weeks and 75% had presented by 10 weeks.

In both the unadjusted and adjusted discrete-time hazards model, any difficulty paying for the abortion/travel was not significantly related to differential time between considering an abortion and presenting at the clinic (Table 3). Incorporating the intersection of difficulty paying for the abortion/travel and ability to cover basic expenses in the past month, those with the lowest means had statistically significantly longer times between considering abortion and presenting at the clinic in both the unadjusted and adjusted models. Specifically, those with the lowest means had 61% lower odds (adjusted hazard odds ratio: 0.39, 95% CI 0.21–0.74) of having an abortion in the next week compared to those with the highest means, given they had not presented at the clinic, all else equal. The alternative model specification dealing with missing data were consistent with the findings reported here (data not shown).

**Table 3:** Modeled odds hazard ratios (OHR and 95% CI) from discrete-time hazards models (n = 164)

	Unadjusted*	Adjusted^†^
Financial variables		
Any difficulty paying (reference no difficulty paying)	0.75 (0.51–1.11)	.75 (0.47–1.20)
Any difficulty paying × ability to cover basic expenses		
Highest means	Ref	Ref
Mixed means	0.98 (0.62–1.54)	0.94 (0.55–1.62)
Lowest means	0.40 (0.22–0.73)	0.39 (0.21–0.74)
In-country care-seeking variables		
Contacted or visited providers in country of residence (reference no in-country care seeking)	0.59 (0.40–0.88)	0.56 (0.36–0.89)
By type of contact˄		
No contact	Ref	Ref
Contacted providers only	0.70 (0.39–1.28)	0.41 (0.18–0.95)
Visited providers	0.55 (0.36–.85)	0.56 (0.34–0.92)

Each variable was included in a separate model.

*Unadjusted models include study country, country of residence, and clinic, ^†^Adjusted models for financial predictors include: study country, country of residence, clinic, age, parity, reported self-managed abortion attempt, prior abortion, travel time, employment status, gestational age at time of survey, had support from family and friends. Adjusted models for in country-care seeking include all those listed under financial model and difficulty paying for the abortion/travel

Highest means represent those who had no difficulty paying for abortion procedure and travel and had funds to cover their basic living expenses in the past month “all the time” or “most of the time.” Mixed means represent those who had some difficulty paying for abortion procedure and travel and had funds to cover basic living expenses in the past month “all the time” or “most of the time.” Those with lowest means had some difficulty paying for abortion procedure and travel and had funds to cover basic living expenses in the past month “sometimes” or “rarely,” or “never.”

Examining the financial implications of travel, those with the lowest means were more likely to report having to delay paying other expenses to fund their abortion (38.7% of respondents) compared to those with mixed means (24.0%) and those with the highest means (2.0%) (Table 4). Thirteen percent of those with the lowest means had to sell something of value, compared to less than 3% of those in the other groups. Those with the highest means primarily reported drawing from their savings (49.0%) or relying on a friend, relative, or partner. Almost 30% of participants with the highest means did not report any measures taken to cover the costs. In fact, almost half of the group with highest means said they did not need to raise funds and those who did have to raise money primarily raised funds within a week. Among those with mixed means, half reported it took up to a week for them to raise the money, and a quarter reported it took them 1–4 weeks. In the group with the lowest means, over 40% took a week or more to raise the funds, with 19.4% reporting it took them over 4 weeks to raise the money for their travel or their abortion procedure. Finally, financial reasons factored more prominently into why those with the lowest means could not get an abortion earlier among those who would have preferred earlier access.

**Table 4:** Cost and financial experiences of abortion and travel among those travelling to England and the Netherlands from European countries with relatively liberal abortion laws (n = 164)

	Have funds for basic expenses all or most of the time		Have funds for basic expenses sometimes, rarely, or never
	No difficulty paying for abortion/travel	Some difficulty paying for abortion/travel	Some difficulty paying for abortion/travel
Traveled alone	17.6%	20.0%	32.3%
Stayed abroad overnight	66.7%	60.0%	58.1%
National insurance not covering any part of abortion cost	88.2%	94.7%	96.8%
Took time off from work to come to appointment	49.0%	65.3%	54.8%
Lost wages because of time off from work*	16.0%	25.0%	23.5%
Special arrangements made for children or other person in care	47.1%	43.8%	48.1%
Actions to cover cost of abortion			
Sold something of value	2.0%	2.7%	12.9%
Delayed/put off paying other expenses	2.0%	24.0%	38.7%
Financial assistance from a friend	3.9%	13.3%	19.4%
Financial assistance from a relative	7.8%	41.3%	35.5%
Financial assistance from a partner	15.7%	33.3%	25.8%
Financial assistance from an abortion fund	0.0%	2.7%	3.2%
Used my savings	49.0%	37.3%	22.6%
Used credit card	3.9%	6.7%	9.7%
Bank gave me credit	0.0%	4.0%	3.2%
Did not report how covered costs	29.4%	2.7%	6.5%
Length of time needed to raise money for cost of travel and abortion			
I didn’t have to raise money	48.0%	12.5%	9.7%
1–< 7 days	44.0%	50.0%	45.2%
1–4 weeks	8.0%	26.4%	22.6%
> 4 weeks	0.0%	6.9%	19.4%
Prefer not to say	0.0%	4.2%	3.2%
Reason could not get an abortion earlier: had issues getting money for abortion^†^	0.0%	8.0%	29.0%
Reason couldn’t get an abortion earlier: had issues getting money for travel^†^	0.0%	4.0%	19.4%

*Proportion of those who had to take time off work

^†^Proportion of those who said they would have preferred to get an abortion earlier (92.5%, 97.5%, and 92.7% respectively)

### Seeking care in country of residence

Among those who did not contact or visit a provider prior to presenting at the clinic in England or the Netherlands, 50% of respondents had presented for care by 2 weeks after having considered abortion compared to 3 weeks among those who contacted or visited an abortion provider in their country of residence (Fig. 1d). In both the unadjusted and adjusted models, having contacted or visited an abortion provider in the country of residence was associated with a longer time to presenting at the clinic abroad for an abortion (Table 3). Among those who had not presented at the clinic abroad at any given week, those who had contacted or visited providers in their country of origin had 44% lower odds (adjusted hazard odds ratio: 0.56, 95% CI 0.36–0.89) of presenting to the clinic abroad in the next week compared to those who had not contacted or visited a provider in their country of residence. Among those who only contacted a provider (versus visiting a provider), the unadjusted hazard odds ratio was not significantly different from the group that did not seek care in their country of residence; however, the effect was significant in the adjusted model (adjusted hazards odds ratio: 0.41, 95% CI 0.18–0.95). The results for those who visited a provider were similar to the main analysis (adjusted hazards odds ratio: .56, 95% CI 0.34-0.92). The sensitivity analyses to address missing data were consistent with the findings reported here (data not shown).

## Discussion

This analysis considered two specific reasons that people seeking abortions outside of their country of residence may be delayed in accessing abortion—financial barriers and abortion care-seeking within a person’s country of residence. Among people who received an abortion outside their country of residence, those who had difficulty paying for an abortion and/or the travel and had insufficient means to cover basic living costs were more delayed in presenting at a clinic abroad for care than those who consistently had enough money to cover basic living costs, regardless of whether they had difficulty paying for the cost of the abortion or travel. Additionally, those who sought in-person care at an abortion provider in their country of residence were significantly more delayed in presenting at a clinic abroad for care compared to those who only contacted a provider or who did not seek abortion care at all in their country of residence.

Among pregnant people travelling abroad for abortion services, the cost of travel and the procedure may be associated with delays in care for those who face less financial security. This is in line with previous research that shows that offering access to abortion without ensuring associated costs are covered restricts who is able to access services and the timeliness with which they are able to do so [33–35]. Governments in France, Belgium, and Italy cover the costs of abortion procedures performed in their own countries by law [36, 37]. In Germany, financial coverage for abortion services in country is based on income thresholds [12]. Despite these varying commitments to cover financial costs of abortion care for care sought within their own borders, countries generally do not pay for care sought abroad. In the United States, a large body of literature documents the impact that lack of insurance coverage has on abortion access and the wellbeing of individuals and families. Lack of insurance coverage compels low-income persons to raise money for the procedure in ways that risk their health and wellbeing by forgoing essentials such as food and electricity, increasing financial instability, and delaying and restricting access to services [38–40]. Our results add to this literature—participants in our study relied on postponing or forgoing payments for other expenses, selling valuable possessions, and leaning on support networks to help finance their travel and procedure. Given that many people who travel for abortion services from European countries with broad legal grounds for abortion in the first trimester but restricted access thereafter are later in pregnancy, there is an urgency to remove gestational age limits to center health equity and to enact policies to ensure access to timely abortion care is guaranteed. These results also speak to the need for organizations (e.g., abortion funds, practical support organizations, or clinic-based funds) to support people financially and logistically seeking abortion care abroad in Europe [41, 42]. Those seeking later abortion services are more often from lower socioeconomic status; compounding costs of travel and the procedure itself may also further restrict who is able to access services [43].

Our findings also point to how interactions with the medical system in a given country of residence may delay people in accessing abortion. In this study, those who had visited a provider in their country of residence were more likely to be delayed in ultimately accessing care abroad. In-country care seeking may have contributed to delays through a number of avenues including difficulty accessing a medical professional with information on how to navigate abortion care access, particularly past the first trimester; and requirements that providers must have extensive and burdensome documentation in order to provide care. These interactions may also require individuals to interact with an objecting provider, mandated counseling, or burdensome waiting periods that push abortion seekers past the legal limit for abortion in their country of residence [7, 16, 44]. During the study period in Germany, for example, the law disallowed abortion providers from legally disseminating or publicizing information on abortion services [45]. While the law has recently been amended to allow providers to list the availability of services online, detailed information may still be limited. Additionally, past the first trimester, many countries require approval from at least one, and in many cases multiple clinicians [28]. Somewhat paradoxically, participants in our study also reported medical professionals to be a key source of information about abortion services [46]. As such, the medical system may act both to delay access to care for some, and as a valuable source of information for others [47, 48].

It is important to note that delays observed in this study among those who sought care in person at an abortion provider in their home country may not have been due to the medical system or providers, but may have been the result of having to organize logistics for care seeking more than once or due to differential access to information on abortion care and laws in their country of residence. However, this would still suggest that gestational age limits and other barriers intersect with interactions with the medical system to create further delays to care. Assessing and improving the resource and information landscape for people seeking abortion may help people get care more quickly. It is important to note that additional factors may delay people in accessing abortion care abroad including support available from friends, family, or partners; difficulty deciding about the abortion; and the gestational age of the pregnancy.

There are a number of limitations to note in this analysis. First, the sample size for the analysis is small—a product of the declining number of people travelling for abortion from relatively liberal settings to the England and the Netherlands. The decline may be due to changing dynamics in the increasing availability of medication abortion self-management [49, 50] or may be related to greater availability of clinic-based abortion in countries of origin, decreased overall demand for abortion, or to changing political arrangements in Europe, most notably the vote for Brexit which had not yet been implemented at the time of the study but that factored into ongoing public dialogue and perceptions [36]. The size of the sample likely limits our power to detect small differences in delays to care. Regardless, small studies using time-to-event modeling are powered to detect larger effects [51, 52] and this study is unique in the population that it includes. Little research focuses on people from European countries where abortion is available on broad grounds in the first trimester travelling abroad for abortion care. Second, only those who were ultimately able to access abortion services abroad were captured in the sample—excluding those who may have wanted an abortion but were unable to travel and those who received an abortion in their country of residence. To measure financial insecurity, we relied on self-assessed relative acute measures instead of income or asset-based measures. While this helps account for differences across contexts, personal spending patterns and individual assessments of sufficient funds are subjective [53]. Future work could extend this work to measure socioeconomic status using income or wealth. Our work is strengthened, however, by the secondary measures of cost and financial experiences that we stratified by the perceived measures of socioeconomic status. Finally, while we tried to capture potential ways in which missing data may have influenced our results, we cannot rule out the possibility that the exclusion of participants with missing data could have influenced the results.

## Conclusions

This paper explores financial and medical system barriers faced by residents of countries in Europe where abortion is available on broad grounds in the first trimester seeking abortion care outside of their country of residence. The findings point to inequities in access to timely abortion care based on socioeconomic status and delays related to seeking care in the country of residence. These findings suggest that policies which govern when (i.e., gestational age limits) and how to have an abortion intersect with health care systems and social stratification to potentially result in differential access in timing of abortion services.

## Data Availability

Due to our commitment to protect the confidentiality and anonymity of those who received abortion services at the participating clinics, we cannot make the data used for this study publicly available for download. The data that support the findings of this study are available on request from the corresponding author.

## Footnotes

1 At the time of study, abortion in France was available on request only until 12 weeks of pregnancy. France modified the law to extend gestational age limits to 14 weeks in 2022 [13].

2 In our recruitment materials for this study, we used the word “women” to describe the population of interest to the study. We did not, however, ask about gender identity in the process of recruitment, nor did we survey participants on their gender identity. Because we cannot report on our participants gender identity and we acknowledge that not all people who are capable of pregnancy and/or desiring of abortion identify as women we will primarily use the gender-inclusive term “people” to refer to and acknowledge the experiences of women and any other pregnant people who participated in our study. We will use gender-specific terms (e.g., “women”) when reporting the results of other research studies, where participants identified as such.
